# Therapeutic Opportunities with Pharmacological Inhibition of CD38 with Isatuximab

**DOI:** 10.3390/cells8121522

**Published:** 2019-11-26

**Authors:** Thomas G. Martin, Kathryn Corzo, Marielle Chiron, Helgi van de Velde, Giovanni Abbadessa, Frank Campana, Malini Solanki, Robin Meng, Helen Lee, Dmitri Wiederschain, Chen Zhu, Alexey Rak, Kenneth C. Anderson

**Affiliations:** 1 Hematology/Oncology, University of California San Francisco, San Francisco, CA 94143-0324, USA; Tom.Martin@ucsf.edu; 2 Sanofi Oncology, Cambridge, MA 02142, USA; Kathryn.Corzo@sanofi.com (K.C.); Helgi.VandeVelde@sanofi.com (H.v.d.V.); Giovanni.Abbadessa@Sanofi.com (G.A.); Frank.Campana@sanofi.com (F.C.); Malini.Solanki@sanofi.com (M.S.); Robin.Meng@sanofi.com (R.M.); Helen.Lee@sanofi.com (H.L.); Dmitri.Wiederschain@sanofi.com (D.W.); Chen.Zhu@sanofi.com (C.Z.); 3 Translational and Experimental Medicine, Sanofi Research & Development, 94403 Vitry-sur-Seine, France; Marielle.Chiron@sanofi.com; 4 Integrated Drug Discovery, Sanofi Research & Development, 94403 Vitry-sur-Seine, France; Alexey.Rak@sanofi.com; 5 Dana-Farber Cancer Institute, Boston, MA 02215, USA

**Keywords:** multiple myeloma, anti-CD38 therapy, isatuximab

## Abstract

CD38 is a transmembrane glycoprotein with ectoenzymatic activity involved in regulation of migration, signal transduction, and receptor-mediated adhesion. CD38 is highly expressed on various malignant cells, including multiple myeloma (MM), and at relatively low levels in other tissues, making it a suitable target for therapeutic antibodies. Several anti-CD38 therapies have been, or are being, developed for the treatment of MM, including daratumumab and isatuximab (SAR650984), respectively. Studies have shown that anti-CD38 therapies are effective in the treatment of relapsed/refractory MM and are well tolerated, with infusion reactions being the most common side effects. They can be used as monotherapy or in combination with immunomodulatory agents, such as pomalidomide, or proteasome inhibitors to potentiate their activity. Here we examine isatuximab and several anti-CD38 agents in development that were generated using new antibody engineering techniques and that may lead to more effective CD38 targeting. We also summarize trials assessing these antibodies in MM, other malignancies, and solid organ transplantation. Finally, we propose that further research on the mechanisms of resistance to anti-CD38 therapy and the development of biomarkers and new backbone regimens with CD38 antibodies will be important steps in building more personalized treatment for patients with MM.

## 1. Introduction

The protein CD38 is a type II transmembrane glycoprotein that plays a role in regulation of migration, signal transduction, and receptor-mediated adhesion by interaction with CD31 or hyaluronic acid [1,2,3]. Furthermore, CD38 serves as an ectoenzyme with adenosine diphosphate -ribose (ADPR) cycling and hydrolase activity, catalyzing the metabolism of two distinct calcium messengers, cyclic ADPR (cADPR) [4,5] and nicotinic acid adenine dinucleotide phosphate (NAADP), respectively [6]. 

Under normal conditions, CD38 is expressed at relatively low levels on myeloid and lymphoid cells and in some non-hematopoietic tissues [1]. In contrast, normal plasma cells and multiple myeloma (MM) cells have high levels of CD38 expression, making CD38 an attractive target for therapeutic antibodies to treat MM [7]. Several CD38-targeting therapies in different phases of clinical development will be described further in this review. Daratumumab (Janssen Pharmaceuticals, Horsham, PA, USA) is a CD38 monoclonal antibody approved for monotherapy use and in combination with anti-MM therapies [8,9]. Isatuximab (formerly SAR650984, Sanofi, Cambridge, MA, USA), which targets a specific epitope on CD38, is under phase 3 clinical assessment and will be the primary focus of this review. Additional CD38 monoclonal antibodies in clinical development will be briefly discussed in Section 5. 

CD38 antibodies kill tumor cells via Fc-dependent immune effector mechanisms including complement-dependent cytotoxicity (CDC), antibody-dependent cell-mediated cytotoxicity (ADCC), antibody-dependent cellular phagocytosis (ADCP), and apoptosis [7,8,10]. The last three mechanisms are dependent on the interaction of the Fc region of the antibody, with Fcγ receptors (FcγRs) expressed on immune effector cells [7]. Notably, due to the differences in epitopes of the CD38-targeting antibodies, their effectiveness to induce CDC, ADCC, ADCP, or apoptosis varies [11]. Isatuximab is the only CD38 antibody that is capable to induce direct apoptosis; daratumumab, MOR202, and TAK-079 induce apoptosis upon secondary cross-linking [7,8,12]. The CD38 antibodies may also improve host antitumor immunity by the elimination of regulatory T cells, regulatory B cells, and myeloid-derived suppressor cells [7].

## 2. Preclinical Data and Mechanisms of Action of Isatuximab

### 2.1. Isatuximab Mechanism of Binding

**Figure 1:**
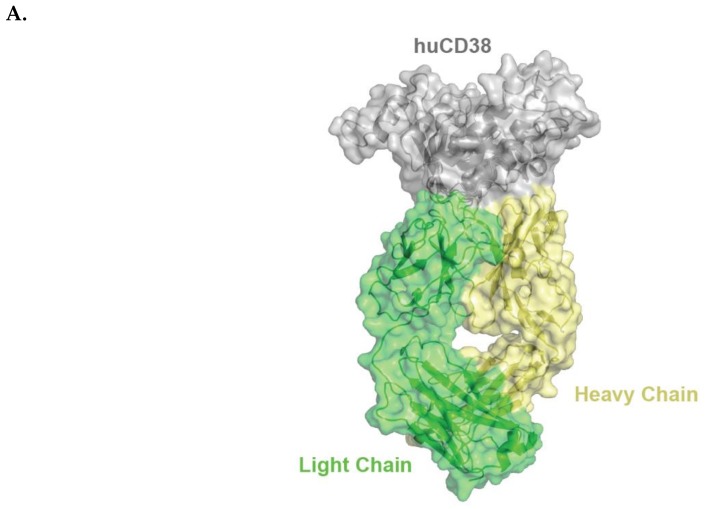

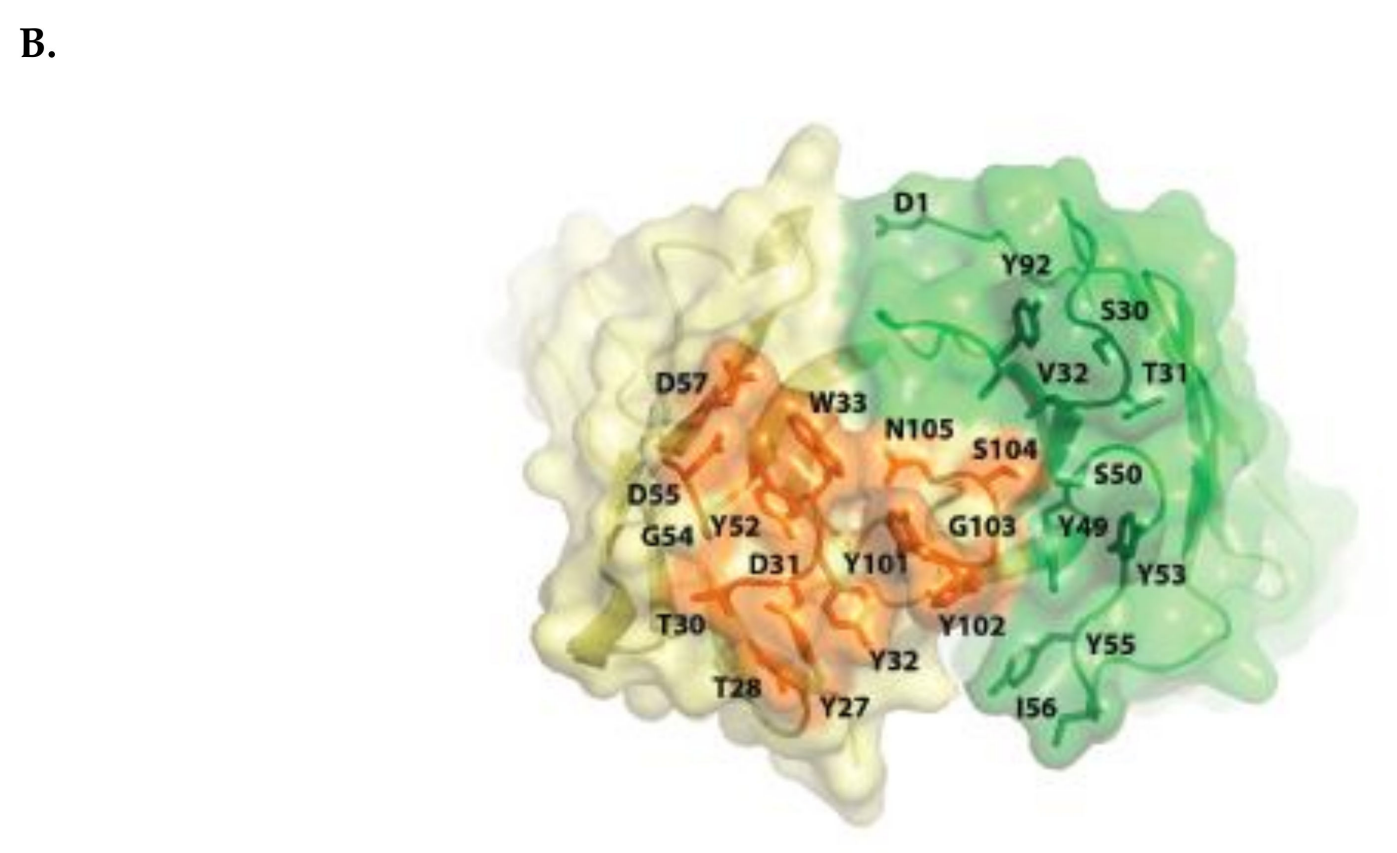
Schematic representation of huCD38/isatuximab-Fab complex (**A**). huCD38 is colored in gray, light chain and heavy chain of isatuximab in green and yellow, respectively. Schematic representation of the paratope of the complex huCD38/isatuximab-Fab (**B**). Light-chain and heavy-chain of isatuximab are colored in green and pale yellow, respectively. The residues part of the paratope are represented in sticks and colored in dark green if located on the light-chain or in orange if located on the heavy-chain of isatuximab. huCD38, human CD38.

Isatuximab binds to a specific discontinuous epitope on CD38 that includes amino acids located opposite to the catalytic site of CD38 [13]. Mapping of the epitope and identification of the paratope were achieved by determining the crystal structure of soluble human CD38 in complex with the Fab fragment from isatuximab at 1.53 Å resolution (Figure 1A; Protein Data Bank identification code: 4 CMH). It is worth noting that isatuximab interacts with CD38 mostly via its heavy-chain paratope comprising 16 amino acids (Tyr27, Thr28, Thr30, Asp31, Tyr32, Trp33, Tyr52, Gly54, Asp55, Asp57, Asp100, Tyr101, Tyr102, Gly103, Ser104, Asn105), while isatuximab’s light-chain paratope includes 10 amino acids (Asp1, Ser30, Thr31, Val32, Tyr49, Ser50, Tyr53, Tyr55, Ile56, Tyr92) (Figure 1B). In this instance, the paratope is defined as antibody residues with atoms within 4.0 Å of the antigen atoms.

Although significant conformational changes are observed in CD38 upon binding by isatuximab, the overall configuration of key residues involved in the CD38 ADP-ribosyl cyclase enzymatic activity is maintained and the catalytic site remains accessible. Since isatuximab strongly inhibits CD38 enzymatic activity, it is likely an allosteric antagonist. This mode of binding is different from other CD38 antibodies such as HB7, which binds at a different location in the C-terminal portion of the CD38 extracellular domain and does not inhibit CD38 enzymatic activity. The isatuximab binding mode appears unique, since other CD38 antibodies do not show or are less potent at inhibiting enzymatic activity of CD38 [13]. Specifically, we have compared epitopes recognized by isatuximab, based on the previously discussed crystal structure, with those recognized by daratumumab, as determined by biochemical method (constrained peptide approach, PepScan) [14].

**Figure 2:**
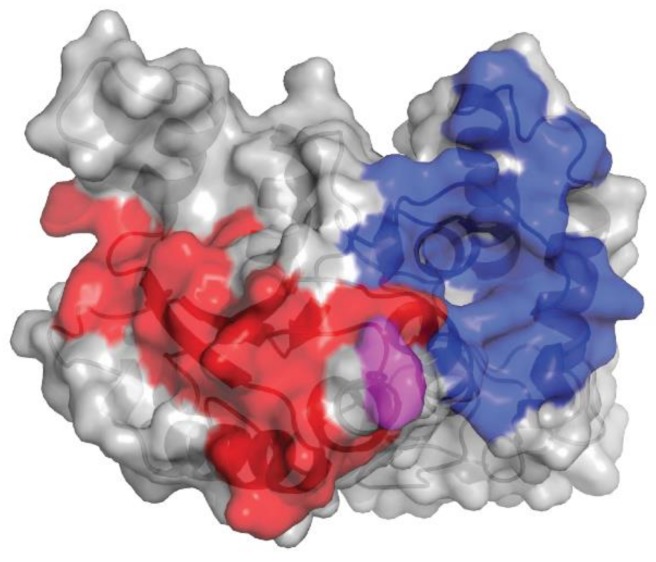
Comparison of isatuximab and daratumumab epitopes on huCD38. The gray shading denotes huCD38, the blue shading denotes the epitope of huCD38 for isatuximab, and the red shading denotes the epitope of huCD38 for daratumumab. Glu233 is highlighted in pink. huCD38, human CD38.

The epitopes of human CD38 (huCD38) interacting with isatuximab and daratumumab are distinct. Isatuximab and daratumumab recognize 23 and 27 amino acids of huCD38, respectively (Figure 2). The Glu233 residue has a highly flexible sidechain, as evident from the poor quality of electron density maps. This residue faces the N-terminal residue Asp1 of isatuximab light chain, also found to be highly flexible. As a result, the interaction between huCD38 Glu233 and isatuximab light-chain Asp1 is likely much weaker than the other interactions found between huCD38 and isatuximab. Differences in physical binding of isatuximab and daratumumab to huCD38 may contribute to the distinct mode of action of each drug.

### 2.2. Isatuximab Uniquely Inhibits CD38 Enzymatic Activity

CD38 has both ADP ribosyl-cyclase activity and hydrolase enzymatic activity (conversion of nicotinamide adenine dinucleotide [NAD] to cADPR and ADPR in CD38-expressing cell lines) [15]. Daratumumab was shown to partially inhibit the cyclase activity of CD38 in vitro using recombinant CD38 protein or CD38-expressing cells [8]. 

**Figure 3:**
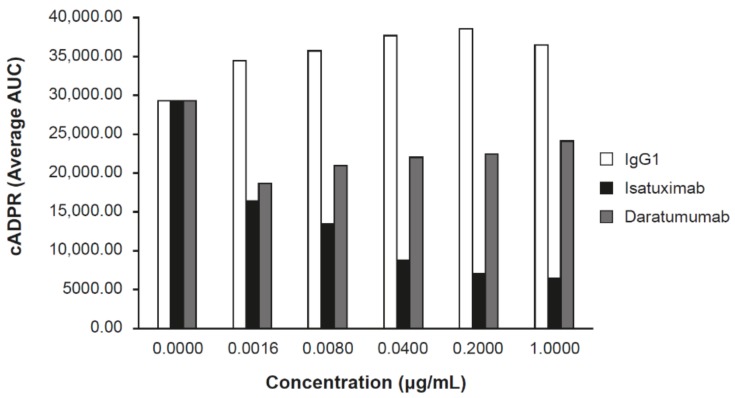
Isatuximab inhibits CD38-NADase activity. MM LP-1 cells cultivated in culture media were treated with the indicated concentrations of IgG1 (control), isatuximab, or daratumumab. Synthesis of cADPR was measured by mass spectrometry. AUC, area under the curve; cADPR, cyclic adenosine diphosphate-ribose; Ig, immunoglobulin; MM, multiple myeloma; NAD, nicotinamide adenine dinucleotide.

Inhibition of the ADP ribosyl-cyclase enzymatic activity of huCD38 protein by isatuximab was evaluated in vitro. Isatuximab almost completely inhibited the enzymatic function of 5 nmol/L recombinant CD38 at concentrations of 20–200 nmol/L [13]. Additionally, isatuximab treatment of CD38-expressing cell lines induced inhibition of CD38 enzymatic activity (Figure 3). Importantly, isatuximab was shown to also inhibit the hydrolase activity of CD38. Treatment of CD38-positive LP-1 MM cell line with isatuximab in time-course and dose-response experiments resulted in inhibition of the synthesis of cADPR, as measured by mass spectrometry. Increasing isatuximab concentrations led to a dose-dependent inhibition of NAD conversion to cADPR (Figure 3). Treatment of LP-1 cells with daratumumab under the same experimental conditions resulted in a limited inhibition of cADPR synthesis with no dose response (Figure 3). CD38 plays a critical role in NAD^+^ degradation and adenosine generation [15]. Elevated adenosine levels have been detected in the bone marrow of MM patients, which may contribute to the overall immunosuppressive microenvironment [16]. A review by Horenstein and colleagues suggested that CD38 may be the dominant enzyme for adenosine generation in diseased bone marrow due to compromised CD39/CD73 function under acidic pH conditions that exist in MM bone marrow niche [16]. Therefore, inhibition of CD38 enzymatic activity may alleviate immunosuppressive microenvironment in bone marrow niche of MM patients. However, establishing clear contribution of adenosine pathway signaling to the anti-tumor activity of anti-CD38 antibodies has been confounded by technical challenges of directly measuring adenosine levels in patients. 

Another feature that may be specific to the CD38 epitope targeted by isatuximab is CD38-induced internalization [17]. Continuous exposure to isatuximab does not result in decreasing CD38 receptor expression in H929, MM1S, OPM2, and RPMI-8226 MM cell lines. In other studies, isatuximab has been shown to induce internalization of CD38 but not its significant release from the MM cell surface [17]. Meanwhile, daratumumab treatment results in the clustering of CD38 molecules into polar aggregates, leading to the release of CD38 in microvesicles [18].

### 2.3. Isatuximab Induces Tumor Cell Death Through Effector Functions

Binding of isatuximab to CD38 on MM cells triggers multiple mechanisms that can lead to the death of the target cancer cells. Some of these mechanisms are mediated by the Fc portion of the antibodies that bind to the FcγRs expressed on effector natural killer (NK) cells and macrophages to trigger ADCC and ADCP, or that allow the fixation of the human complement to trigger CDC.

#### 2.3.1. ADCC

ADCC is considered the main mechanism of isatuximab-induced tumor cell death as supported by several lines of experimental evidence. For example, in one study, isatuximab was shown to mediate potent ADCC activity through purified human NK cells against 15 tumor cell lines, with maximum lysis of 30%–90% of target cells and half maximal effective concentration (EC_50_) of 0.2–8.0 ng/mL [13]. ADCC activity by isatuximab against different MM cell lines is shown in Table 1. Likewise, isatuximab triggered ADCC against primary tumor plasma cells derived from 13 patients, with significant tumor cell depletion observed in vitro (*p* = 0.003) [17].

Isatuximab can also enhance the anti-MM activity of standard therapies, including proteasome inhibitors like bortezomib and carfilzomib, and immunomodulatory drugs such as pomalidomide and lenalidomide. In vitro experiments demonstrate that the combination of isatuximab and pomalidomide results in enhanced direct cytotoxicity and lysis of CD38-positive MM cells by ADCC compared with that of isatuximab alone. The pretreatment of peripheral blood mononuclear cells with 2 µM pomalidomide increased isatuximab-induced ADCC of MM1S MM cells from below 40% to above 80%. In six patient-derived MM cell lines, isatuximab-induced cytotoxicity was significantly increased in the presence of pomalidomide (*p* < 0.001) [12]. 

#### 2.3.2. ADCP

ADCP of antibody-opsonized cancer cells occurs through binding to FcγRs, specifically via the low-affinity receptors FcγRIIA and FcγRIIIA. Isatuximab was shown to mediate ADCP in the presence of human macrophages against Ramos cells at 10 μg/mL, to a similar extent as rituximab, a monoclonal antibody that binds to the cell surface protein CD20 [13]. Isatuximab induced ADCP with 60% phagocytosed Ramos cells, compared with 25% in untreated samples, with an EC_50_ value of 5 ng/mL [13]. Additionally, isatuximab was shown to trigger ADCP only in the RPMI-8226 MM cell line with high CD38-receptor density (RD; median 43%, *p* = 0.005), although nonsignificant ADCP against H929, MM1S, and OPM2 MM cell lines with low CD38 RD was observed [17].

#### 2.3.3. CDC

Isatuximab was shown to induce strong CDC in the presence of human serum in Raji and Daudi cell lines, with activity similar to rituximab [13]. CDC activity was observed in 7 of 15 blood cancer cell lines evaluated, with up to 90% maximum lysis and EC_50_ values varying widely from 8 to 230 ng/mL [13]. Among MM cell lines LP-1, MOLP-8 and NCI-H929 that have high CD38 RD (790,000 to 233,000; [13]), isatuximab-induced CDC was observed in LP-1 and MOLP-8, with percentages of cell lysis of 82% and 62%, and corresponding EC_50_ values of 0.18 and 1.53 nM (27.3 and 228.2 ng/mL), respectively. However, in RPMI8226, H929, MM1S, and OPM2 MM cell lines, which have low CD38 RD, isatuximab-mediated CDC was not induced, based on the absence of C3 deposition and impact on cell survival [17]. 

### 2.4. Isatuximab Induces Direct Apoptosis

Isatuximab was selected in an antibody screen for further evaluation based on its ability to directly trigger MM cell death in the absence of cross-linking agents and independent of effector cells [12,13]. Daratumumab and TAK-079 lack the ability to directly induce MM cell death [11]; however, FcγR-mediated cross-linking of daratumumab induces programmed cell death of CD38-positive MM tumor cell lines [10]. By comparing daratumumab efficacy in a syngeneic in vivo tumor model using Fcγ-chain knockout mice or NOTAM mice (transgenic mice expressing physiological levels of signaling-inactive FcγRs), the authors found that the inhibitory FcγRIIb, as well as activating FcγRs, induce daratumumab cross-linking–mediated programmed cell death [10].

The pro-apoptotic activity of isatuximab in the absence of cross-linking agents was seen in MOLP-8 MM cell lines, which have high levels of CD38 RD (790,000 molecules/cell) [13]. This ability of isatuximab to induce apoptosis was also tested in primary cells isolated from bone marrow aspirates of seven patients with MM. Isatuximab noticeably increased the percentages of Annexin V–positive cells over background levels in MM samples tested, with a mean increase of 25% Annexin V–positive cells [13].

Isatuximab induced direct cytotoxicity without cross-linking in a dose-dependent manner in p53 mutated or deleted MM cell lines (RPMI8226, U266, JJN3) that correspond to unfavorable MM subgroups, which were transduced to overexpress CD38 [12]. In MOLP-8 cells, isatuximab also induced cytotoxic response, and the coculture with bone marrow stromal cells (BMSCs) did not abrogate isatuximab-induced cytotoxicity.

Isatuximab triggers both the caspase-dependent apoptotic pathway and the lysosome-mediated cell death pathway in MM cells. Isatuximab was shown to induce reactive oxygen species production, which occurs downstream of lysosomal activation and contributes to MM cell death. These direct effects are independent of Fc fragment binding, supplementing the classical Fc-dependent killing mechanisms via effector cells [12].

### 2.5. Activity of Isatuximab in Mouse Tumor Models

In vivo activity of isatuximab was assessed in subcutaneous xenograft models derived from MOLP-8 and NCI-H929 MM cell lines [13]. In the MOLP-8 model, isatuximab was well tolerated and active at 40, 25, and 15 mg/kg when administered twice weekly for three weeks, with treated-to-control (T/C) values (relative tumor volume measurements) of 8%, 10%, and 12%, respectively. According to National Cancer Institute (NCI; Bethesda, MD) standards, T/C values ≤42% correspond to active and T/C values ≤12% correspond to highly active. Similarly, in the NCI-H929 model, isatuximab treatment was well tolerated and highly active at 40, 20, and 10 mg/kg administered twice weekly for three weeks. At the end of the study, 10/10 tumor-free survivors and 9/10 tumor-free survivors were observed for the 10-mg/kg and 5-mg/kg regimens, respectively [13].

**Figure 4:**
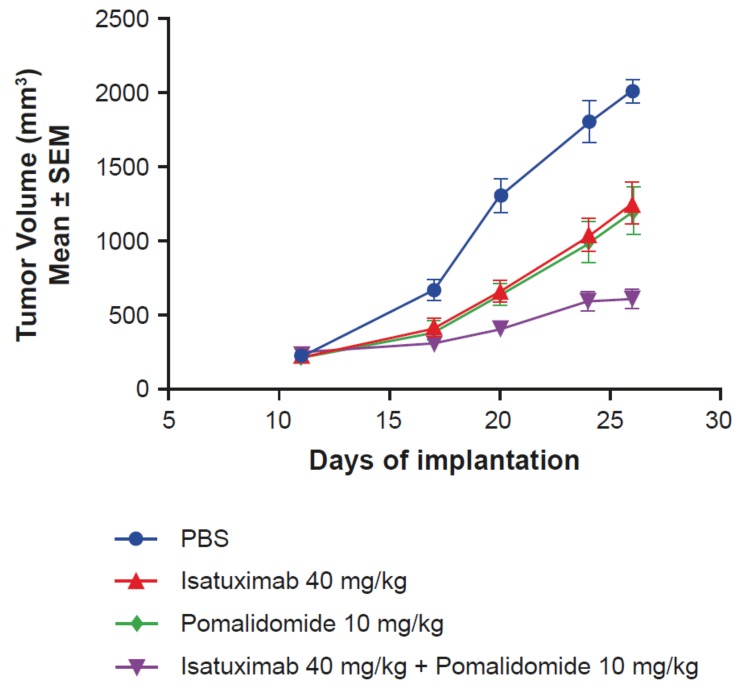
Pomalidomide enhances isatuximab activity in vivo. Female NSG mice (*n* = 8/group) with subcutaneously implanted MOLP-8 tumors (3 × 10^6^ cells in 50% matrigel) were treated with PBS, isatuximab (5 doses of 40 mg/kg IV), pomalidomide (10 mg/kg intraperitoneally, once daily for 14 days), or the combination of both isatuximab and pomalidomide. IV, intravenously; NSG, NOD scid gamma; PBS, phosphate-buffered saline; SEM, standard error of mean.

Due to the previously demonstrated synergistic effects of pomalidomide on isatuximab-induced ADCC of MM cells in vitro [12], in vivo experiments using an MOLP-8 MM mouse xenograft model were designed. The combination of isatuximab and pomalidomide resulted in enhanced antitumor activity (T/C value of 22%) compared with the activity of isatuximab (T/C value of 56%) and pomalidomide alone (T/C value of 46%), without affecting body weight (Figure 4). Taken together, these results demonstrate that pomalidomide increases isatuximab activity in vitro and in vivo.

### 2.6. Isatuximab and Daratumumab Possess Several Mechanistic Differences 

In vitro studies described above uncovered several mechanistic differences between isatuximab and daratumumab, likely resulting from different binding epitopes on CD38. Daratumumab-induced ADCC can be augmented by lenalidomide and bortezomib, whereas isatuximab and pomalidomide demonstrate synergistic ADCC induction both in vitro and in vivo (Figure 3) [12]. Isatuximab demonstrates potent direct killing activity based on a greater increase in apoptosis of CD38-expressing cancer cells [12]. Moreover, isatuximab led to a dose-dependent inhibition of CD38 enzymatic activity, while daratumumab under the same experimental conditions produced a more limited inhibition without obvious dose response (Figure 3).

## 3. Targeting CD38 in MM

MM is a neoplasm characterized by the accumulation of monoclonal malignant plasma cells in the bone marrow. Asymptomatic plasma cell dyscrasias, such as monoclonal gammopathy of undetermined significance (MGUS) and smoldering MM, have a propensity to progress to symptomatic MM [19]. Cytogenetic analyses have shown that MM is a heterogenous disease, with two main primary chromosomal events: (i) hyperdiploid MM presents with several trisomies (chromosomes 3, 5, 7, 9, 11, 15, 19, and 21), and (ii) non-hyperdiploid MM is associated with primary Ig heavy-chain translocations, such as t(4;14), t(11;14), t(14;16), or t(14;20), which result in overexpression of specific oncogenes [20]. During further disease evolution, myeloma cells acquire secondary chromosomal aberrations, including (i) amplification of chromosome 1q, (ii) deletion of chromosome 1p, (iii) deletion of chromosome 17p, which includes the tumor-suppressor gene *TP53*, and (iv) translocations involving the *MYC* locus on chromosome [20,21,22]. The International Myeloma Working Group (IMWG) classifies the presence of del(17p) and/or t(4;14), and/or t(14;16) as high-risk cytogenetic markers associated with reduced survival of patients with MM [23]. More recently, amplification of chromosome 1q and t(4;14) has been classified as intermediate-risk MM [24].

MM is the second most frequent hematologic malignancy after lymphoma, accounting for 1% of all cancers and approximately 10% of all hematologic malignancies [25,26]. Worldwide in 2016 there were 138,509 incident cases of MM with 98,437 estimated deaths [27]. Incident cases from 1990 to 2016 increased by 126% globally, with an age-standardized incidence rate of 2.1 cases per 100,000 in 2016 [27]. The median age of patients with MM at diagnosis is approximately 70 years, and only 37% of patients display an age <65 years [28]. The median survival of patients with relapsed MM has increased from 12 months before 2000 to 24 months after 2000, due to the introduction of effective treatments including autologous stem-cell transplantation (ASCT) [29]. Availability of modern therapies, such as immunomodulatory drugs and proteasome inhibitors, have further prolonged the survival rates of patients with MM [29], with a median overall survival of approximately 5 years [26]. Prognosis of relapsed/refractory MM (RRMM) patients remains poor, however, and novel therapeutic approaches are urgently needed [22]. 

Despite available treatments, studies have shown that the depth of response decreases with each additional line of therapy [30,31,32]. MM remains incurable with patients continuously relapsing over time, and is associated with significant patient burden. In this context, CD38 represents a promising therapeutic target. MM treatment with CD38 antibodies in clinical trials has been extensively reviewed by others [22,33]; here we review relevant isatuximab clinical findings.

### 3.1. Isatuximab Monotherapy Activity in MM

Isatuximab has been investigated as monotherapy for the treatment of RRMM in a phase 1/2 trial (ClinicalTrials.gov, NCT01084252) [34,35,36]. Martin and colleagues [35] evaluated isatuximab as monotherapy against RRMM in a phase 1 dose-escalation/expansion study, with the goal to determine the maximum tolerated dose of isatuximab. Overall, 84 patients with RRMM progressing on or after standard therapy received 0.0001–20 mg/kg of isatuximab intravenously (IV) weekly or every 2 weeks. Following dose escalation, two expansion cohorts (ECs; EC1: standard-risk and high-risk patients; EC2: only high-risk patients) were added at 10 mg/kg every 2 weeks. High-risk RRMM was defined as abnormal genotype (del[17p], 1q amplification, t[4;14], or t[14;16]), disease relapse within 6 months of ASCT, or high-risk gene-expression profile defined by investigator. Four patients with RRMM were treated in the accelerated dose-escalation cohorts, 36 in the basic dose-escalation cohorts, 37 in the expansion-phase cohorts (EC1, *n* = 19; EC2, *n* = 18), and 7 in the 20-mg/kg weekly cohort. Patients had received a median of 5 (range 1–13) prior lines of therapy, and 62% had received prior carfilzomib or pomalidomide. The maximum tolerated dose of isatuximab was not reached, since no cumulative adverse events (AEs) were noticed. The most frequent AEs were grade 1 and 2 infusion reactions (IRs), occurring in 51% of patients. In patients receiving isatuximab ≥10 mg/kg, the overall response rate (ORR) was 23.8%, including one complete response. In high-risk patients treated with isatuximab 10 mg/kg weekly or biweekly, the ORR was 16.7%. The median duration of response at doses ≥10 mg/kg was 25 (range 8–30) weeks among high-risk patients versus 36 (range 6–85) weeks for other patients. The authors concluded that isatuximab demonstrated a manageable safety profile and clinical activity in patients with RRMM [35].

The phase 2 dose-finding study confirmed that isatuximab monotherapy is active and generally well tolerated in heavily pretreated RRMM [34,36]. In stage 1, a total of 97 patients with RRMM who were previously treated with at least three lines of therapy or refractory to immunomodulatory drugs and proteasome inhibitors were randomized to the following isatuximab regimens: (i) 3 mg/kg biweekly, (ii) 10 mg/kg biweekly × 2 cycles of 28 days then every four weeks, (iii) 10 mg/kg biweekly, or (iv) 20 mg/kg weekly × 4 doses then biweekly [36]. Patients received a median of 5 (range 2–14) prior lines of therapy, with 86%, 61%, 80%, 57%, or 88% refractory to lenalidomide, pomalidomide, bortezomib, carfilzomib, or immunomodulatory drugs plus proteasome inhibitors, respectively. IRs occurred in 49% of patients (mostly grades 1 and 2), with 94% occurring during the first infusion. Most common AEs were nausea (33%), fatigue (30%), dyspnea (26%), and cough (24%), typically grades 1 or 2. As of November 2015, the median treatment duration was 13.1 weeks, and 22 patients remained on treatment. The ORR for the four dosing schemes were 9%, 20%, 29%, and 24%, respectively. The dose of 20 mg/kg weekly × 4 doses then biweekly was chosen for stage 2 of the study [36]. In stage 2, patients were randomized on a 2:1 ratio to receive either isatuximab (20 mg/kg weekly, then every 2 weeks) or isatuximab combined with dexamethasone (40 mg/day [20 mg/day in patients ≥75 years old]) [34]. Among 165 patients included in the analysis, the ORR was 26% with isatuximab monotherapy and 44% with combination therapy. Furthermore, addition of dexamethasone improved the median PFS by 4.4 months. The safety profile in both arms was similar, with grade ≥3 AEs occurring in 20% of patients treated with isatuximab monotherapy and 18% for the isatuximab plus dexamethasone arm. IRs occurred in 40% of patients and 4% of patients discontinued treatment due to IRs [34]. While, isatuximab monotherapy is active and generally well tolerated in RRMM patients, dexamethasone may have a synergistic effect with isatuximab.

Currently, two trials are evaluating the efficacy and tolerability of isatuximab monotherapy for the treatment of RRMM in Chinese patients (ClinicalTrials.gov, NCT03733717) and Japanese patients (ClinicalTrials.gov, NCT02812706). Initial results from the phase 1/2 study in Japanese patients confirmed the 20 mg/kg QW/Q2W dosing regimen for isatuximab monotherapy in this patient population [37]. Further, isatuximab monotherapy was well tolerated and favorable efficacy was observed with an ORR of 36.4% in patients who received isatuximab 20 mg/kg QW/Q2W.

Additionally, isatuximab is being assessed in a phase 1 trial (ClinicalTrials.gov, NCT02514668) as monotherapy in patients with RRMM, including those previously treated with daratumumab. Finally, isatuximab is being investigated for the treatment of patients with high-risk smoldering myeloma to prevent progression to active MM in a phase 2 trial (ClinicalTrials.gov, NCT02960555). See Table 2 for a list of all trials currently evaluating isatuximab as a monotherapy in patients with MM.

### 3.2. Combination Therapy for MM with Isatuximab

Since MM is a heterogeneous cancer with several subclones presenting different susceptibility to different agents, combination therapy has been a successful approach to improve clinical outcomes [20]. Based on the efficacy of CD38 antibodies, these agents are very attractive as a component of combination therapy regimens. In the phase 1/2 dose-finding study (ClinicalTrials.gov, NCT01084252), there was no clear difference in response rate between 10 and 20 mg/kg doses [36]. The 20 mg/kg dose was selected for monotherapy based on pharmacokinetics and pharmacodynamics analyses. Subsequent studies have determined 10 mg/kg as an appropriate dose for combination therapy.

#### 3.2.1. Isatuximab in Combination with Immunomodulatory Drugs

The combination of CD38 monoclonal antibodies with immunomodulatory drugs, such as lenalidomide and pomalidomide, have a synergistic effect and resulted in increased ADCC [12,38,39,40]. Two phase 1b dose-escalation trials studied the combination of isatuximab with lenalidomide/dexamethasone (ClinicalTrials.gov, NCT01749969) [41] and with pomalidomide/dexamethasone (ClinicalTrials.gov number, NCT02283775) [42] in RRMM patients. 

In the phase 1b lenalidomide/dexamethasone combination study, patients with RRMM were treated with isatuximab in two dose schedules: (i) 3, 5, or 10 mg/kg every other week or (ii) 10 or 20 mg/kg weekly for 4 weeks and then every 2 weeks thereafter, in combination with lenalidomide 25 mg (days 1–21) and dexamethasone 40 mg (weekly) [41]. Fifty-seven patients with a median of 5 prior lines (range 1–12), including 83% refractory to previous lenalidomide therapy, were treated. Isatuximab in combination with lenalidomide and dexamethasone was generally well tolerated, with only one grade 3 dose-limiting toxicity (pneumonia) observed with the 20 mg/kg dose schedule, which resolved following treatment discontinuation. At least one grade ≥3 treatment-related AE was recorded in 88% of patients, with 56% experiencing a serious treatment-related AE. The most common isatuximab-related AEs were IRs (56%), which were grade 1/2 in 84% of patients. The ORR was 63% for the 10 mg/kg every other week cohort, 50% for the 10 mg/kg weekly for 4 weeks and every 2 weeks thereafter cohort, and 50% for the 20 mg/kg weekly for 4 weeks and every 2 weeks thereafter cohort. Notably, the ORR was 52% in 42 evaluable lenalidomide-refractory patients. Overall median PFS was 8.5 months (95% CI 4.73–16.59 months). The authors concluded that combining isatuximab with lenalidomide and dexamethasone provided clinical activity and was well tolerated in heavily pretreated patients with RRMM [41].

In the phase 1b combination study with pomalidomide/dexamethasone [42], 45 patients who had received ≥2 prior lines of therapy, including lenalidomide and a proteasome inhibitor, received (i) isatuximab IV at 5 (*n* = 8), 10 (*n* = 31), or 20 (*n* = 6) mg/kg weekly for 4 weeks, followed by every 2 weeks; (ii) pomalidomide 4 mg (days 1–21); and (iii) dexamethasone 40 mg (weekly) in 28-day cycles until progression or intolerable toxicity. Patients received a median of 3 (range 1–10) prior lines of therapy and 91% were refractory to their last line of therapy, with 82% lenalidomide refractory and 84% proteasome inhibitor refractory. Median treatment duration was 9.6 months, and 42% of patients remained on treatment at the time of study analysis. The most common AEs included fatigue (62%), upper respiratory tract infection (42%), and dyspnea (40%); IRs occurred in 42% of patients. The most common grade ≥3 AE was pneumonia, occurring in 17.8% of patients. The most common lab abnormalities were hematologic (lymphopenia, leukopenia, anemia, 98% each; neutropenia, 93%; thrombocytopenia, 84%). The ORR was 62%, with a median duration of response of 18.7 months and a median progression-free survival (PFS) of 17.6 months. The authors concluded that adding isatuximab to pomalidomide/dexamethasone therapy results in significant clinical activity with a manageable safety profile in heavily pretreated patients with RRMM and lead to the initiation of the pivotal ICARIA-MM trial [42].

In the phase 3 randomized, open-label, multicenter international ICARIA trial, the combination of isatuximab with pomalidomide/dexamethasone (Isa-Pd group) was compared with pomalidomide/dexamethasone (control group) in patients with RRMM (ClinicalTrials.gov, NCT02990338) [43,44]. A total of 307 patients with RRMM who received a median of 3 prior lines of therapy, all patients received lenalidomide and a proteasome inhibitor, and were refractory to the last therapy were enrolled. Patients received (i) isatuximab 10 mg/kg IV every week for the first 4 weeks, then every 2 weeks; (ii) pomalidomide 4 mg orally on days 1–21; and (iii) dexamethasone 40 mg (20 mg for patients ≥75 years old) orally or IV every 28 days until progression or unacceptable toxicity. Patients in the control group received pomalidomide and dexamethasone in the same schedule. Patients received a median of 3 (range 2–11) prior lines of therapy, with 92.5% being refractory to lenalidomide, 59% were refractory to lenalidomide at the last line of therapy before study entry, and 75.9% being refractory to a proteasome inhibitor. At median follow-up of 11.6 months, median PFS was 11.5 months in the Isa-Pd group versus 6.5 months in the control group (hazard ratio [HR] 0.596, 95% confidence interval [CI] 0.44–0.81, *p* = 0.001). An independent review committee, blinded from the treatment arm, was implemented to ensure consistency in the assessment of disease response and without bias and used central lab data for M-protein and central imaging review. A PFS benefit was maintained across all preplanned subgroups, including patients >75 years old, patients with renal function impairment, patients with high-risk cytogenetics, patients who received >3 prior lines of therapy, and patients refractory to lenalidomide and a proteasome inhibitor and refractory to lenalidomide in the last line. The ORR was 60.4% for the Isa-Pd group versus 35.3% for the control group (*p* < 0.0001). The very good partial response rate or better was 31.8% for the Isa-Pd group versus 8.5% for the control group (*p* < 0.0001), and minimal residual disease negativity at a threshold of 10^–5^ was reported in 5.2% of patients treated with the Isa-Pd versus 0% in the control group (intent-to-treat [ITT] population). At analysis cut-off date (October 11, 2018), overall survival was immature (99 events), but a trend to overall survival improvement in the group treated with Isa-Pd was observed versus the control group (HR 0.687, 95% CI 0.461–1.023). Median treatment duration was 41 weeks in the Isa-Pd group versus 24 weeks in the control group. Isatuximab IRs were reported in 38.2% of patients, with 2.6% of those being grade 3–4. AEs grade ≥3 were observed in 86.8% of patients treated with Isa-Pd versus 70.5% of patients treated with control. The most common grade ≥3 AEs were infections (42.8% in the Isa-Pd group vs 30.2% in the control) and the most common laboratory abnormality was neutropenia (84.9% in patients treated with Isa-Pd vs 70.1% in the control). Addition of isatuximab significantly improved PFS and ORR of patients with RRMM versus pomalidomide/dexamethasone, with a manageable safety profile [45].

#### 3.2.2. Isatuximab in Combination with Proteasome Inhibitors 

In a phase 1b dose-escalation trial (ClinicalTrials.gov, NCT02332850), the safety and efficacy of combining isatuximab with the proteasome inhibitor carfilzomib in RRMM was assessed [46,47]. Patients received three isatuximab dosing levels IV: (i) 10 mg/kg biweekly (*n* = 3), (ii) 10 mg/kg weekly for four doses (*n* = 24), and (iii) 20 mg/kg weekly for four doses (*n* = 6) followed by biweekly doses in combination with standard carfilzomib (27 mg/m^2^: days 1, 2, 8, 9, 15, 16, every 28 days). Thirty-three patients with a median of three prior lines of therapy (range 2–8) were treated across the three dosing levels. All patients received prior immunomodulatory drugs and bortezomib, and 45% received prior carfilzomib, and 79% were double-refractory. An ORR of 60.6% was seen at all isatuximab dose levels, with 24% of patients achieving very good partial response or better. No dose-limiting toxicities or severe AEs were observed, with the most frequent serious grade 3 AE being hypertension (9%). Grade 1 and 2 IRs occurred in 48% of patients, but all completed therapy. It was determined that combining isatuximab and carfilzomib is well tolerated and shows encouraging anti-RRMM activity [46,47].

Isatuximab is currently being evaluated in combination with carfilzomib and dexamethasone in patients with RRMM who have been previously treated with 1–3 lines of therapy (IKEMA; ClinicalTrials.gov, NCT03275285).

See Table 3 for a list of all trials currently evaluating isatuximab in combination with other therapies, including immunomodulatory agents, proteasome inhibitors, and other compounds, in patients with MM.

### 3.3. Isatuximab in Patients with Newly Diagnosed MM

Based on the efficacy and a manageable safety profile of the CD38-targeting antibodies in patients with RRMM, these agents are currently also being investigated as earlier lines of therapy and in patients with newly diagnosed MM (NDMM).

Several studies are currently evaluating the role of isatuximab as earlier lines of therapy in patients with NDMM (Table 3). These include two trials in the first-line setting with bortezomib, lenalidomide, and dexamethasone backbone therapy. The first is the GMMG HD7 study assessing isatuximab in combination with bortezomib, lenalidomide, and dexamethasone, followed by maintenance with lenalidomide, in transplant-eligible patients with NDMM (ClinicalTrials.gov, NCT03617731). The IMROZ study is evaluating isatuximab in combination with bortezomib, lenalidomide, and dexamethasone in transplant-ineligible patients with NDMM (ClinicalTrials.gov, NCT03319667). Isatuximab is also being evaluated in combination with carfilzomib, lenalidomide, and dexamethasone in high-risk patients with NDMM, stratified by transplant eligibility (ClinicalTrials.gov, NCT03104842). Lastly, isatuximab is being assessed in combination with either bortezomib, cyclophosphamide, and dexamethasone or bortezomib, lenalidomide, and dexamethasone in patients with NDMM who are not eligible for, or elect not to receive transplantation (ClinicalTrials.gov, NCT02513186). Interim analysis from both arms of this trial have been reported [48,49].

Patients with NDMM ineligible for transplantation were treated in an induction phase with either isatuximab 10 mg/kg or 20 mg/kg weekly (cycle 1), followed by every 2 weeks (cycles 2–12); bortezomib (1.3 mg/m^2^) for cycles 1–12; cyclophosphamide (300 mg/m^2^) for cycles 1–12; and dexamethasone (20 mg/day) for cycles 1–12, followed by a maintenance phase of 4-week cycles that included isatuximab at the assigned dose and dexamethasone (20 mg) on day 1 for all cycles [48]. The maintenance phase was followed by an isatuximab 10 mg/kg dose expansion phase. As of August 31, 2017, 17 patients had been treated with isatuximab in combination with bortezomib, cyclophosphamide, and dexamethasone (eight in dose-escalation [10 mg/kg, n = 4; 20 mg/kg, n = 4], nine in dose-expansion). Median duration of exposure was 12 months (range 0.2–22.9). Four patients discontinued treatment, two due to AEs, one due to disease progression, and one patient withdrew consent; 13 (76.5%) patients remained on treatment. Isatuximab doses were withheld in seven patients, and doses of cyclophosphamide, bortezomib, and dexamethasone were withheld in nine, 11, and 12 patients, respectively. No dose-limiting toxicities were reported and the maximum tolerated dose was not reached. Treatment-related AEs of any grade occurred in 100% of patients, with grade ≥3 AEs being reported in 76.5% of patients. Serious AEs were reported in five patients (39%) (bronchospasm, gastroenteritis, and dorsal pain [grade 3, each]; mesenteric vein thrombosis [grade 2]; and sudden death [grade 5]). IRs were observed in 47% patients, with one patient reporting a grade 3 IR; all others were grade 1/2 in severity. The ORR was 93% in 15 evaluable patients, including 73% with a very good partial response rate or better. The data suggest that isatuximab in combination with bortezomib, cyclophosphamide, and dexamethasone is tolerated in patients with NDMM, and based on the encouraging results, the study was expanded to include a isatuximab, bortezomib, lenalidomide, and dexamethasone cohort [48].

In the expansion cohort, patients were treated in two phases: (i) an induction phase of four 6-week cycles that included isatuximab (10 mg/kg) weekly (cycle 1), followed by every 2 weeks (cycles 2-4); bortezomib (1.3 mg/m^2^) for cycles 1–4; lenalidomide (25 mg/day) for cycles 1–4; and dexamethasone (20 mg/day), and (ii) a maintenance phase of 4-week cycles that included isatuximab (10 mg/kg) every 2 weeks (all cycles); lenalidomide (25 mg/day) for all cycles; and dexamethasone (40 mg or 20 mg for patients >75 years of age) for all cycles [49]. As of March 22, 2018, 22 patients who received ≥1 dose of isatuximab were included in the safety analysis and the first 14 patients who completed the four induction cycles were included in the preliminary efficacy analyses. Median age was 71 (range 63–77) years and the median number of cycles was 5.5 (range 1–9). Three patients discontinued treatment, two due to AEs and one patient withdrew consent; 19 (86%) patients are continuing treatment. Dose reduction of bortezomib, lenalidomide, and dexamethasone was required in 29%, 16%, and 28% of patients, respectively. AEs of any grade occurred in 86% of patients, with grade ≥3 AEs being reported in 46% of patients. Grade ≤2 IRs were observed in 63% patients, with one patient reporting a grade 3 IR. The median time to first response was 1.4 months and no patient has progressed (median follow-up, 7.49 months). Minimal residual disease negative status was achieved in 38.5% of patients. The ORR was 93% with all but one responder showing very good partial response rate or better. These interim data demonstrate that the combination of isatuximab with bortezomib, lenalidomide, and dexamethasone is well tolerated and shows promising results in NDMM patients [49].

## 4. Investigation of CD38 Antibodies in Other Therapeutic Areas

### 4.1. Other Malignancies

As CD38 is expressed on the surface of numerous immune cells, including those of B-lymphocyte, T-lymphocyte, and myeloid origin [8], there is scientific rationale for the use of CD38 therapies in other hematologic malignancies including Hodgkin’s lymphoma (HL), diffuse large B-cell lymphoma (DLBCL), and peripheral T-cell lymphoma (PTCL) [50,51]. Therefore, CD38 monoclonal antibodies have the potential to benefit patients with a variety of malignancies.

Both daratumumab and isatuximab were shown to have activity against lymphoma and leukemia cell lines and xenograft mouse models [13,14,52]. Daratumumab has been shown to induce disruption of chronic lymphocytic leukemia (CLL) adhesion and migration [53] and ADCC in a panel of 10 NK-cell–T-cell lymphoma (NKTCL) cell lines [54]. Moreover, daratumumab induces a cytotoxic effect in Waldenstrom macroglobulinemia (WM) cells, but results were variable and cell-line dependent, with ADCC and not CDC was shown to be the prominent mechanism of action [55].

Despite these pre-clinical findings, readouts from clinical studies have shown unfavorable results to date. A phase 2 trial (ClinicalTrials.gov, NCT02413489) of daratumumab in relapsed/refractory B-cell non-Hodgkin’s lymphoma (NHL) subtypes, including mantle-cell lymphoma (MCL), DLBCL, and follicular lymphoma (FL), showed that this CD38 antibody is not effective as monotherapy in NHL [56]. ORR was not evaluable in MCL patients, 6.7% in DLBCL patients, and 12.5% in FL patients [56]. Another phase 2 trial (ClinicalTrials.gov, NCT02927925) is testing the efficacy of daratumumab monotherapy for patients with nasal-type relapsed/refractory NKTCL [57]. Interim results showed that daratumumab demonstrated an ORR of 35.7% and did not meet prespecified futility criteria in patients with relapsed/refractory NKTCL [57]. Similarly, a trial testing isatuximab as monotherapy in recurrent/refractory acute lymphoblastic leukemia (ALL) or T-lymphoblastic lymphoma (ClinicalTrials.gov, NCT02999633) was terminated due to an unsatisfactory benefit-risk ratio (as specified in the protocol). Therefore, CD38 antibodies may need to be combined with other agents to successfully treat leukemias and lymphomas. As such, the use of isatuximab is being evaluated in combination with standard chemotherapies in the treatment of pediatric patients with ALL or acute myeloid leukemia (AML) in a phase 2 trial (ClinicalTrials.gov, NCT03860844; Table 4).

The use of isatuximab as monotherapy for the treatment of refractory/recurrent systemic light-chain amyloidosis (AL) is being tested in a phase 2 trial (ClinicalTrials.gov, NCT03499808; Table 4). Daratumumab is being evaluated in combination with cyclophosphamide, bortezomib, and dexamethasone in newly diagnosed systemic light-chain AL in a phase 3 trial (ClinicalTrials.gov, NCT03201965), and as monotherapy in previously untreated patients with Stage 3B light-chain AL (ClinicalTrials.gov, NCT04131309). 

CD38 therapies are also being explored in a number of solid tumors. Daratumumab monotherapy is being investigated in patients with metastatic renal cell carcinoma (mRCC) or muscle-invasive bladder cancer (ClinicalTrials.gov, NCT03473730). Both daratumumab and isatuximab are being studied in combination with checkpoint inhibitors in solid tumors, such as prostate cancer, non-small cell lung cancer (NSCLC), squamous cell carcinoma of the head and neck (SCCHN), ovarian cancer, and liver cancer.

Immune checkpoint blockade removes inhibitory signals of T-cell activation, enabling tumor-reactive T cells to overcome regulatory mechanisms that normally block autoimmunity and effectively eliminate cancerous cells [58]. Immunosuppressive checkpoints, like cytotoxic T lymphocyte–associated protein-4 (CTLA-4) and the blockade of programmed cell death-1 (PD-1) and its ligands (PD-L1/PD-L2), have provided a successful treatment target in immunotherapy. The distribution and expression of these proteins, however, vary by cell and malignancy type [58]. Data obtained in preclinical tumor models suggest tumor-associated CD38 elicits resistance to the PD-1/PD-L1 blockade through the inhibition of CD8-positive T-cell activity, as was observed in an NSCLC in vivo model, thus explaining the observed significant efficacy of a PD-L1 inhibitor used in combination with CD38 antibodies [59,60]. These findings form the rationale for multiple ongoing clinical trials investigating the use of checkpoint inhibitors in combination with daratumumab (reviewed by van de Donk and colleagues [33]) or isatuximab in a number of malignancies, including solid tumors (Table 4). Isatuximab is being evaluated in combination with checkpoint agents such as cemiplimab (anti-PD-1) and atezolizumab (anti-PD-L1). The combination of isatuximab with cemiplimab in the treatment of classic HL (cHL), DLBCL, and PTCL is being studied in a phase 1/2 trial (ClinicalTrials.gov, NCT03769181). The same combination is being evaluated in a phase 1/2 trial (ClinicalTrials.gov, NCT03367819) for the treatment of metastatic castration-resistant prostate cancer (mCRPC) patients who are naive to anti-PD-1/PD-L1 therapy and NSCLC patients who progressed on prior anti-PD-1/PD-L1 therapy. This patient population was chosen based on prior preclinical studies that demonstrated that tumors with high levels of T-cell infiltration increased expression of CD38, a novel mechanism of acquired resistance to immune checkpoint therapy [59,60]. Isatuximab in combination with atezolizumab (anti-PD-L1) is being studied for the treatment of platinum-refractory recurrent/metastatic SCCHN, platinum-resistant/refractory epithelial ovarian cancer (EOC), recurrent glioblastoma multiforme (GMB), and hepatocellular carcinoma (HCC; ClinicalTrials.gov, NCT03637764). Two other phase 1/2 trials are investigating the efficacy and safety of atezolizumab (anti-PD-L1) combined with multiple immunotherapy-based therapy combinations, including with isatuximab, for the treatment of (i) mCRC that became refractory to first- and second-line standard therapies (ClinicalTrials.gov, NCT03555149) and (ii) locally advanced or metastatic urothelial carcinoma (UC) that has progressed during or following a platinum-containing regimen (ClinicalTrials.gov, NCT03869190).

### 4.2. Solid Organ Transplantation

Alloantibody-producing plasma cells express CD38 at a higher level than other CD38-positive hematopoietic cells (such as monocytes, NK cells, and B-cell progenitors) and, most importantly, comparable to that of MM cells [17,61,62]. Therefore, CD38 is a rational target for depleting plasma cells that produce harmful alloantibodies, with potential clinical utility in solid-organ transplantation as a means for desensitization and/or treatment of antibody-mediated rejection (AMR).

For patients with end-stage renal disease, transplantation is associated with decreased mortality, better quality of life, and lower expenditures compared with chronic dialysis treatment [63,64]. Human leukocyte antigen (HLA)–sensitized patients have prolonged wait times for transplantation and may never find a compatible donor, depending on the extent of sensitization. This limitation is attributed to the presence of HLA antibodies that significantly enhance the risk of rapid rejection of a new organ and therefore reduce a candidate’s transplant eligibility [65,66]. Desensitization therapy aims to overcome the humoral incompatibility by removing or reducing the presence of reactive antibodies, thereby increasing the chance of finding compatible or acceptable donors for transplantation.

Currently, there is no approved or standard therapy for desensitization in patients awaiting kidney transplantation or for treatment of AMR. The most common desensitization protocols involve plasmapheresis, IVIG, and/or rituximab, which do not target the terminally differentiated antibody-secreting plasma cells responsible for alloantibody production in the bone marrow, and consequently are often associated with a quick antibody rebound effect [67,68].

To date, only subjective clinical reports have been published on the use of CD38 antibodies in desensitization and treatment of AMR. Kwun and colleagues reported the use of daratumumab in desensitization in a heart transplant candidate [69]. Multiple courses of plasmapheresis, high-dose IVIG, and rituximab result in no significant changes in HLA antibody levels (calculated Panel Reactive Antibody score [cPRA] 98%), with deterioration of clinical condition. Following 8 weekly injections of daratumumab, the patient’s HLA antibody levels significantly decreased (cPRA score 62%), subsequently enabling heart transplantation. The decrease in HLA antibody was associated with high depletion of peripheral CD38-positive plasma cells, suggesting target engagement. Another study has also reported the successful treatment of resistant AMR with daratumumab in a highly sensitized HLA-incompatible kidney transplant recipient [70].

CD38 agents are also under investigation for desensitization in patients awaiting solid-organ transplantation. Stanford University has initiated a phase 1 clinical trial (ClinicalTrials.gov, NCT04088903) to study the use of daratumumab in this population.

## 5. Anti-CD38 Agents in Development

Along with daratumumab and isatuximab, there are many anti-CD38 compounds in development (Table 5) for treatment of MM and other indications discussed previously. CD38 monoclonal antibodies in late phases of clinical development are discussed briefly below.

### 5.1. Daratumumab

Daratumumab was selected from a panel of 42 antibodies, based on its ability to induce CDC in Daudi cells [14]. Additionally, daratumumab induced CDC in freshly isolated MM cells obtained from the bone marrow of 13 previously untreated or refractory patients with MM, as well as in XG-1 MM cells in the presence of BMSCs [14]. Daratumumab also eliminates MM cells via ADCC and ADCP via antibody binding to activating FcγRs on immune effector cells [14,52]. Furthermore, FcγR-mediated cross-linking of daratumumab induces programmed cell death of CD38-positive MM tumor cell lines [10]. In a combined ADCC/CDC in vitro assay, daratumumab and a biosimilar isatuximab antibody reduced NK cell numbers [71]. Further studies are necessary to decipher the potential impact of NK cell reductions on the activity of monoclonal anti-CD38 therapies.

Daratumumab was approved in November 2015 by the US Food and Drug Administration (FDA) and in May 2016 by the European Medicines Agency (EMA) to be used as monotherapy for the treatment of RRMM [33]. A Biologics License Application (BLA) was submitted to the FDA in July 2019 seeking approval for a new subcutaneous (SC) formulation of daratumumab in RRMM patients [72]. This application was based on the results of the phase 3 COLUMBA trial (ClinicalTrials.gov, NCT03277105) [73]. SC daratumumab is co-formulated with recombinant human hyaluronidase PH20 (rHuPH20) using the Halozyme ENHANZE^®^ drug delivery technology (Halozyme, San Diego, CA). The COLUMBA trial was a noninferiority comparison between the FDA-approved intravenous formulation of daratumumab with the SC formulation in RRMM patients. In this randomized, open-label, multicenter study, a total of 522 RRMM patients were randomized to receive the SC formulation (n = 263; 1,800 mg daratumumab + rHuPH20 [2,000 U/mL]) versus the intravenous arm (n = 259; 16 mg/kg) weekly for C1-2 (28-day cycles), every 2 weeks for C3-6, and every 4 weeks thereafter. At a median follow-up of 7.5 months, the ORR was 41% for the SC formulation compared to 37% for the intravenous daratumumab. SC daratumumab retained at least 89% of the benefit of the intravenous formulation (97.5% confidence). Median PFS was 5.6 months versus 6.1 months for daratumumab SC versus intravenous (HR, 0.99; 95% CI, 0.78–1.26). A comparable safety profile was observed with the SC formulation versus the intravenous, with significant lower rate of IRs (12.7% versus 34.5%, respectively; *P* = 0.0001). The authors concluded that SC daratumumab is non-inferior to the intravenous formulation of daratumumab [73]. 

### 5.2. MOR202

MOR202 is a HuCAL derived human IgG1 CD38 monoclonal antibody that is under development in China only. MOR202 induces ADCC and ADCP, but not CDC [74]. Cytotoxicity of MOR202 was synergistically enhanced in vitro and in vivo by bortezomib and lenalidomide [75,76]. An ongoing phase 1/2a open-label, dose-escalating study in RRMM (ClinicalTrials.gov, NCT01421186) showed that MOR202 was well tolerated with mainly hematological toxicity in doses up to 16 mg/kg [77]. Interim analysis reported that IRs (grade 1) were seen in only one out of 17 patients [78]. Responses were reported in six out of 15 evaluable patients: two very good partial responses and four partial responses, with five out of six still ongoing. The longest duration of response was > 10 months in a patient receiving MOR202 monotherapy. All nine patients receiving MOR202 monotherapy derived clinical benefits. In conclusion, MOR202 showed excellent infusion tolerability and overall safety profile [78].

### 5.3. TAK-079

TAK-079 is a cytolytic immunoglobulin (Ig) G1 lambda CD38 monoclonal antibody that is being investigated as an SC formulation [79]. The agent received Orphan Drug Designation from the US Food and Drug Administration (FDA) in January 2019. In vitro studies with human cell lines demonstrated that binding of TAK-079 to CD38 induced depletion of human B-cell lines by ADCP and CDC; in most cases, cell lines with increased CD38 expression were more susceptible to cell lysis [80].

Top-line results for a randomized phase 1 trial in healthy participants (ClinicalTrials.gov, NCT02219256) that assessed the safety, pharmacokinetics, and pharmacodynamics of IV or SC injection of TAK-079 indicated that the agent is a potent and convenient next-generation anti-CD38 therapy [81]. The maximum doses administered within the phase 1 trial were 0.06 mg/kg IV and 0.6 mg/kg SC. Following a 2-hour IV infusion, mean maximum serum concentration (C_max_) was 100.4 ng/mL, while the mean C_max_ following a single SC injection was 23.0 ng/mL, which was observed 24 hours post dose and decreased gradually to below the limit of quantification (median of 8 days). The mean area under the Receiver Operating Characteristics curve (AUROC) for AUROC_last_ and AUROC_∞_ values were 90.4 and 212 ng*day/mL, respectively. Pharmacodynamic effects included a transient, dose-dependent reduction in NK cells (at ≥0.003 mg/kg IV), about 167-fold less than the lowest dose reported for NK-cell reduction by daratumumab at dose ≥0.5 mg/kg administered to patients with RRMM. In each participant dosed at 0.6 mg/kg SC, >90% reduction of plasmablasts was observed. This generally peaked 2 days after injection and returned to baseline levels by 29 days. AEs were mild or moderate and the agent was well tolerated, with no safety concerns identified [81].

A phase 1/2a study (ClinicalTrials.gov, NCT03439280) is currently assessing the safety and tolerability, maximum tolerated dose, and recommended phase 2 dose of TAK-079, both as monotherapy and when combined with a backbone regimen of pomalidomide/dexamethasone in patients with RRMM. Another phase 1b study (ClinicalTrials.gov, NCT03984097) is determining the recommended phase 2 dose of TAK-079 when administered to participants with NDMM in combination with the backbone treatment regimens of lenalidomide/dexamethasone or bortezomib/lenalidomide/dexamethasone.

## 6. Future Directions

### 6.1. Mechanisms of Resistance to CD38 Antibodies

Although CD38 antibodies have been clinically proven as an effective therapy for MM, both primary and acquired resistance have been reported [7]. Primary resistance to daratumumab or isatuximab has been linked to CD38 RD [17,82,83]. Acquired resistance has been linked to a reduction in CD38 expression levels on MM cells within hours after starting daratumumab treatment, which was associated with protection against ADCC and CDC [82,84]. These effects, however, were also observed in patients with deep and durable responses, thus excluding CD38 reduction alone as a mechanism of daratumumab resistance [84]. At the time of progression during daratumumab therapy, selection of daratumumab-resistant MM cells with high expression of the complement-inhibitory proteins CD55 and CD59 was observed [82].

The presence of high-risk cytogenetic abnormalities, such as t(4;14), t(14;16), or del17p, is associated with reduced survival of patients with MM and with CD38 antibody resistance [7]. In the randomized phase 3 POLLUX (ClinicalTrials.gov, NCT02076009) and CASTOR (ClinicalTrials.gov, NCT02136134) studies, the addition of daratumumab to lenalidomide/dexamethasone or bortezomib/dexamethasone markedly improved the outcome of high-risk patients versus those receiving lenalidomide/dexamethasone or bortezomib/dexamethasone alone [85,86]. High risk, conferred by the presence of t(4;14), t(14;16), or del17p, however, was not completely abolished by adding daratumumab [7,87]. Moreover, the median PFS of patients with MM treated with daratumumab plus pomalidomide/dexamethasone was poorer in high-risk patients versus standard-risk patients (3.9 vs 10.3 months), while ORR was similar in both groups [88]. Moreover, two large phase 3, randomized, first-line trials evaluating the addition of daratumumab to standard induction therapies including daratumumab combined with: (i) lenalidomide and dexamethasone (MAIA; ClinicalTrials.gov, NCT02252172), and (ii) bortezomib, melphalan and prednisone (ALCYONE; ClinicalTrials.gov, NCT02195479 demonstrated favorable results with the addition of daratumumab to standard induction therapies in subgroup analyses of untreated MM patients with high-risk disease, although interpretation of these results is limited due to the small number of patients [89,90]. A third large phase 3, randomized, first-line trial evaluating the addition of daratumumab to bortezomib, thalidomide and dexamethasone (CASSIOPEIA; ClinicalTrials.gov, NCT02541383) did not show a greater response in the daratumumab group versus the bortezomib, thalidomide, and dexamethasone group in untreated MM patients with high-risk disease [91]. These results are confounded by the fact that high-risk patients have characteristics associated with poor response and require more dosing modifications and reductions during treatment, resulting in lower exposure rates that further hinder response. While high-risk patients treated with isatuximab had a median duration of response of 25 (range 8–30) weeks versus 36 (range 6–85) weeks for other patients in a phase 1 monotherapy trial [35], in the phase 3 ICARIA trial, the observed PFS benefit in the Isa-Pd group was maintained across patients with high-risk cytogenetics and was similar to patients with standard risk cytogenetics (HR 0.66 [0.33–1.28 95% CI] and HR 0.62 [0.42–0.93 95% CI], respectively). Although high-risk MM patients benefit from CD38 antibodies, the poor-risk cytogenetic abnormalities might have a negative impact on clinical outcome in patients across treatments, and further research is needed in these populations.

The development of drug antibodies may neutralize the activity of CD38 antibodies; yet, neutralizing anti-daratumumab and anti-isatuximab antibodies have not been detected in treated patients to date [7]. A better understanding of molecular and biochemical mechanisms that cause differential therapeutic efficacy and resistance toward CD38 antibodies may contribute to further optimization and individualization of treatments that overcome resistance.

### 6.2. Predictive Biomarkers for CD38 Antibodies

Predictive biomarkers can help determine appropriate patient populations for better, individualized treatment options with CD38 therapies. Prognostic biomarkers could also be beneficial in predicting response to CD38 antibodies, further improving and personalizing treatment. Apart from the assessment of CD38 RD on MM cells, there are currently no biomarkers with a predictive value of clinical response to CD38 antibody treatments. 

Casneuf and colleagues [92] used a broad aptamer-based proteomics platform (SomaSCAN™; SomaLogic, Inc., Boulder, CO, USA) to evaluate 1129 clinical serum samples before and after 8 weeks of treatment with daratumumab 16 mg/kg to identify proteins associated with clinical response to the antibody. The investigators found 51 proteins to be significantly different between clinical responders and nonresponders, including tumor necrosis factor subfamily 8 (TNFSF8/CD30L), TNFSF9/CD137L, macrophage stimulating 1 (MST1), interleukin-1B (IL1B), cadherin (CDH) 1, and CDH3 [92].

Atanackovic and colleagues [93] performed immunophenotyping of four patients with MM being treated with isatuximab enrolled in a phase 1 clinical study (NCT02514668). They found that two patients who had little to no preexisting antibody responses at baseline did not develop any new antibody responses during isatuximab treatment. Two patients with antibody responses at baseline clinically responded to isatuximab treatment and also demonstrated new or increasing autologous IgG antibody responses against tumor-associated antigens: the first patient against melanoma-associated antigen (MAGE)-C2, the second against New York esophageal squamous cell carcinoma 1 (NY-ESO-1) and MAGE-C1. The authors also observed a newly developed T-cell response against CD38 after these two patients started isatuximab therapy, and additionally against NY-ESO-1 in the second patient. Both antibody-positive patients had also developed polyclonal T-cell responses against CD38. Importantly, the authors did not observe any new or increasing autologous antibody responses against MM-associated antigens in the two other patients who had not clinically responded to isatuximab, indicating that antigen-specific immune responses may be used as biomarkers to predict response to anti-CD38 therapies [93].

Further investigations are necessary to characterize biomarkers to predict the activity of CD38 antibodies and personalize treatment.

### 6.3. New Backbone Regimens with Anti-CD38 Therapies 

CD38 antibodies have multiple modes of action that provide potent antitumor activity, which can explain their high activity as monotherapy agents in patients with RRMM. Agents from different drug classes with distinct or synergistic mechanisms of action are increasingly being used to reduce the risk of developing drug resistance and induce a deeper response [94]. The efficacy of CD38 antibodies can be enhanced by adding a therapy with a different mode of action, such as immunomodulatory drugs and proteasome inhibitors. Immunomodulatory drugs stimulate NK cells, thereby enhancing antitumor activity by activating the effector cells of ADCC. Proteasome inhibitors may enhance NK cell–mediated cytotoxicity, augmenting anti-CD38-induced cell death.

Isatuximab was shown to be a safe addition to the MM backbone regimens (immunomodulatory drugs, proteasome inhibitors, and glucocorticoids), markedly increasing their efficacy. Combining isatuximab with pomalidomide as a new backbone combination was shown to be effective in an in vivo study (Figure 4).

As previously discussed, CD38 antibodies can also be paired with checkpoint inhibitors such as anti-PD-1 and anti-PD-L1 therapies. This novel combination is being explored in other malignancies (Table 4), as well as in MM (Table 3). The role of the PD-1/PD-L1 pathway in mediating immune escape of malignant plasma cells [95], combined with the fact that PD-L1 is highly expressed on plasma cells isolated from patients with MM but not on normal plasma cells [96,97,98,99], led to an increased interest in the use of anti-PD-1/PD-L1 therapy in MM. Preliminary results of a phase 1 trial of the anti-PD-1 nivolumab used in combination with daratumumab revealed an acceptable safety profile in patients with RRMM [100]. A list of ongoing trials studying the combination of daratumumab with checkpoint inhibitors has been provided by others [22,33]. Likewise, isatuximab in combination with cemiplimab (anti-PD-1) is being evaluated for the treatment of RRMM (Table 3; ClinicalTrials.gov, NCT03194867).

### 6.4. Potential of Combining Immunotherapeutic CD38 Approaches in MM

Monoclonal CD38 antibodies can be used to expand the response to other immunotherapeutic approaches to treat MM, such as chimeric antigen receptor T cell (CAR-T) and CD38/CD3 bispecific T-cell engager (BiTE) therapies. Several CAR-T therapies specific for different MM-associated antigens besides CD38, such as CS1, BCMA, SLAMF7, CD44v6, and CD19, proved to be effective in preclinical models and/or in clinical trials (reviewed by Morandi and colleagues [22] and Franssen and colleagues [101]). Similarly, BiTE therapies designed to link T-cell CD3 with MM-specific receptors besides CD38, such as BCMA, CD19, G-protein–coupled receptor family C group 5 member D (GPRC5D), and the Fc receptor-like protein 5 (FcRH5) have shown good efficacy in preclinical studies and are now being investigated in clinical trials (reviewed by Abramson [102]). Combining monoclonal CD38 antibodies with CAR-T and BiTE therapies may eradicate MM tumor cells more precisely and effectively. 

## 7. Conclusions

Anti-CD38 therapy has shown unprecedented outcomes in MM versus earlier treatment with proteasome inhibitors, immunomodulatory drugs, and chemotherapy. Preclinical and clinical studies indicate CD38 represents a safe target for immunotherapeutic approaches in patients with MM, either as monotherapy or in combination with conventional chemotherapy agents. Ongoing clinical trials continue to assess the efficacy and safety of isatuximab in the treatment of MM and other malignancies.

Both isatuximab and daratumumab induce ADCP and CDC, and both CD38 antibodies demonstrate ADCC activity that can be potentiated by other antitumor agents [12,103]. However, differences in the ability to induce direct apoptosis were observed between isatuximab and daratumumab, with isatuximab demonstrating potent direct killing activity [12]. Moreover, a key distinguishing feature of isatuximab is its capability to inhibit CD38 ectoenzymatic activity [11,12,13,16].

The development of next-generation anti-CD38 therapies with optimized modes of action may lead to more effective CD38 targeting. Further research is needed, however, to better understand anti-CD38 resistance mechanisms. The development of biomarkers and new backbone regimens with CD38 antibodies for the treatment of MM may help optimize and personalize patient treatment options.

## Figures and Tables

**Table 1:** ADCC activity by isatuximab against MM cell lines.

Cell Lines	Maximum NK-Mediated Lysis, %	EC_50_ Values, pM (ng/mL)
LP-1	37	13 (2.02)
MOLP-8	28	1 (0.16)
NCI-H929	27	50 (7.61)

*Abbreviations*: ADCC, antibody-dependent cell-mediated cytotoxicity; EC_50_, half maximal effective concentration; MM, multiple myeloma; NK, natural killer.

**Table 2:** Clinical studies evaluating the efficacy of isatuximab monotherapy in patients with MM.

Clinical Trial	Phase	Patients	Primary Endpoints
NCT02960555 ^a^	2	Smoldering myeloma	Determine the rate of response according to the IMWGC of isatuximab as monotherapy
NCT01084252	1/2	RRMM	Evaluate DLTs and the ORR of isatuximab as monotherapy
NCT02812706	1/2	RRMM	Evaluate DLTs and the ORR of isatuximab in Japanese patients
NCT03733717	1	RRMM	Evaluate the pharmacokinetics, safety, and tolerability of isatuximab in Chinese patients
NCT02514668	1	RRMM	Evaluate safety, tolerability, and the ORR of isatuximab in patients previously treated with daratumumab

^a^ Investigator-sponsored study (M.D. Anderson Cancer Center in collaboration with the National Cancer Institute). Abbreviations: DLTs, dose-limiting toxicities; IMWGC, International Myeloma Working Group Criteria; ORR, overall response rate; RRMM, relapsed/refractory multiple myeloma.

**Table 3:** Clinical studies evaluating the efficacy of isatuximab in combination with other agents in patients with MM.

Clinical Trial	Phase	Patients	Treatment
NCT03319667 (IMROZ)	3	NDMM ineligible for transplant	Isatuximab + VRd vs VRd
NCT03275285 (IKEMA)	3	RRMM, 1–3 prior lines of therapy	Isatuximab + Kd vs Kd
NCT03617731^a^ (GMMG HD7)	3	NDMM eligible for transplant	Isatuximab + VRd, then maintenance with R
NCT03104842^b^	2	NDMM with high-risk cytogenetic profile	Isatuximab + KRd
NCT03194867	1/2	RRMM, ≥3 prior lines of therapy	Isatuximab + cemiplimab
NCT04083898^c^	1/2	Penta-refractory MM	Isatuximab + BPr
NCT01749969	1b	RRMM	Isatuximab + Rd
NCT02332850^d^	1	RRMM, ≥2 prior lines of therapy	Isatuximab + K
NCT02513186	1	NDMM ineligible for transplant	Isatuximab + VCd; Isatuximab + VRd
NCT04045795	1	RRMM	Isatuximab IV + Pd; Isatuximab SC + Pd

^a^ Investigator-sponsored trial (University of Heidelberg Medical Center). ^b^ Investigator-sponsored trial (University Hospital Tuebingen). ^c^ Sponsored by Sanofi in collaboration with Washington University School of Medicine. ^d^ Investigator-sponsored trial (University of California, San Francisco). Abbreviations: B, bendamustine; C, cyclophosphamide; d, dexamethasone; IV, intravenous; K, carfilzomib; MM, multiple myeloma; NDMM, newly diagnosed MM; P, pomalidomide; PD-1, programmed cell death-1; Pr, prednisone; R, lenalidomide; RRMM, relapsed/refractory MM; SC, subcutaneous; V, bortezomib.

**Table 4:** Clinical studies evaluating the efficacy of isatuximab in malignancies other than MM.

Clinical Trial	Phase	Malignancy	Treatment
NCT03860844	2	RR ALL and AML	Isatuximab + standard chemotherapies
NCT03499808 ^a^	2	RR systemic light-chain AL	Isatuximab
NCT03769181	1/2	RR cHL, DLBCL, and PTCL	Isatuximab + cemiplimab (anti-PD-1)
NCT03367819	1/2	mCRPC and NSCLC	Isatuximab + cemiplimab (anti-PD-1)
NCT03637764	1/2	Unresectable HCC, platinum-refractory recurrent/metastatic SCCHN, platinum-resistant/refractory EOC and recurrent GBM	Isatuximab + atezolizumab (anti-PD-L1)
NCT03555149 (Morpheus-CRC) ^b^	1/2	mCRC that became refractory to first- and second-line standard therapies	Atezolizumab (anti-PD-L1) combined with other immunotherapies, including isatuximab
NCT03869190 (MORPHEUS mUC) ^b^	1/2	Locally advanced or metastatic UC that has progressed during or following a platinum-containing regimen	Atezolizumab (anti-PD-L1) combined with other immunotherapies, including isatuximab

^a^ Investigator-sponsored study (Southwest Oncology Group in collaboration with the National Cancer Institute). ^b^ Sponsored by Hoffmann-La Roche. Abbreviations: AL, amyloidosis; ALL, acute lymphoblastic leukemia; AML, acute myeloid leukemia; CLL, chronic lymphocytic leukemia; cHL, classic Hodgkin’s lymphoma; DLBCL, diffuse large B-cell lymphoma; EOC, epithelial ovarian cancer; GBM, glioblastoma multiforme; HCC, hepatocellular carcinoma; mCRC, metastatic colorectal cancer; mCRPC, metastatic castration-resistant prostate cancer; NHL, non-Hodgkin’s lymphoma/leukemia; NSCLC, non-small cell lung cancer; PD-1, programmed cell death-1; PD-L1, programmed cell death-ligand 1; PTCL, peripheral T-cell lymphoma; RR, relapsed/refractory; SCCHN, squamous cell carcinoma of the head and neck; UC, urothelial carcinoma.

**Table 5:** Anti-CD38 agents currently in development.

Agent	Company	Modality	Highest Phase
Daratumumab-rHuPH20 (Dara-SC)	Janssen/Genmab	mAb	BLA
Isatuximab	Sanofi	mAb	BLA
MOR202/TJ202 (MOR03087)	I-Mab/MorphoSys	mAb	3
TAK-079 (SC)	Takeda	mAb	1/2
CAR-T/TCR-T	Shenzhen BinDeBio	Cell therapy	1/2
Multi-CAR-T	Shenzhen Geno Immune	Cell therapy	1/2
TAK-573	Takeda	Immunocytokine	1/2
SAR442085	Sanofi	Fc-engineered	1
TAK-169	Takeda	ETB	1
T-007	Sorrento Therapeutics	Cell therapy	1
AMG 424	Amgen	TCE	1
GBR 1342	Glenmark	TCE	1
Isatuximab (SC)	Sanofi	mAb	1
HexaBody-CD38	Janssen/Genmab	Fc engineered	Preclinical
CD38-ARM (KP1196, KP1237)	Kleo/PeptiDream	ARM	Preclinical
TSK011010/CID103	CASI Pharmaceuticals	mAb	Preclinical
STI-5171	CASI Pharmaceuticals	mAb	Preclinical
Anti-CD38/IGF-1 R bsAb scFV	I’rom Group/GeneTry	bsAb	Preclinical
Anti-CD38 SIFbody	Momenta Pharmaceuticals	Fc engineered	Preclinical
CAR38-MILs	WindMIL	Cell therapy	Preclinical
CD38 DART	Sorrento Therapeutics	Cell therapy	Preclinical
Actinium-225 dara	Actinium Pharmaceuticals	Radionuclide	Preclinical
STI-6129	Sorrento Therapeutics	ADC	Preclinical
Anti-CD38/anti-CD3	IGM Biosciences	TCE	Preclinical
CD38 TCE	Sorrento Therapeutics	TCE	Preclinical
Y-150	Wuhan YZY	TCE	Preclinical

*Abbreviations*: ADC, antibody drug conjugate; ARM, antibody-recruiting molecule; BLA, Biologics License Application; bsAb, bispecific antibody; CAR-T, chimeric antigen receptor T cell; DART, dual-affinity retargeting; ETB, engineered toxin body; mAb, monoclonal antibody; SC, subcutaneous; SIF, selective immunomodulator of Fc receptors; TCE, T-cell engager; TCR-T, T-cell receptor–T cell.
